# Social network analysis of COVID-19 vaccine YouTube videos in Odisha, India: mapping the channel network and analyzing comment sentiment

**DOI:** 10.1186/s12919-023-00260-3

**Published:** 2023-07-07

**Authors:** Neil Alperstein, Paola Pascual-Ferrá, Rohini Ganjoo, Ananya Bhaktaram, Julia Burleson, Daniel J. Barnett, Amelia M. Jamison, Eleanor Kluegel, Satyanarayan Mohanty, Peter Z. Orton, Manoj Parida, Sidharth Rath, Rajiv Rimal

**Affiliations:** 1 Department of Communication, Loyola University, Baltimore, Maryland USA; 2 School of Medical and Health Sciences, George Washington University, WashingtonD.C., USA; 3 Bloomberg School of Public Health, Johns Hopkins University, Baltimore, Maryland USA; 4 Development Corner (D-COR), Bhubaneswar, Odisha India; 5 Wellflix Inc., Hillsborough, USA; 6 Swasthya Plus Network, Bhubaneswar, Odisha India

**Keywords:** COVID-19 vaccine acceptance, Message design, Social network analysis, Sentiment analysis, YouTube recommender system

## Abstract

India has reported more than 35 million confirmed cases of COVID-19 and nearly half a million cumulative deaths. Although vaccination rates for the first vaccine dose are quite high, one-third of the population has not received a second shot. Due to its widespread use and popularity, social media can play a vital role in enhancing vaccine acceptance. This study in a real-world setting utilizes YouTube videos in Odisha, India where the platform has deep penetration among the 18–35 target population, and secondarily their family and peers. Two contrasting videos were launched on the YouTube platform to examine how those videos operate within the broader recommender and subscription systems that determine the audience reach. Video analytics, algorithms for recommended videos, visual representation of connections created, centrality between the networks, and comment analysis was conducted. The results indicate that the video with a non-humorous tone and collectivistic appeal delivered by a female protagonist performed best with regard to views and time spent watching the videos. The results are of significance to health communicators who seek to better understand the platform mechanisms that determine the spread of videos and measures of viewer reactions based on viewer sentiment.

## Introduction

More than 315 million cases have been recorded and more than 5.5 million people have died worldwide due to the COVID-19 pandemic [1]. Facing the second-largest burden of COVID-19 disease, India has reported more than 35 million confirmed cases and nearly half a million cumulative deaths [2]. India’s COVID-19 vaccination campaign, the world’s largest vaccination program, was initiated in early 2021. The objective of the campaign was to fully immunize all 1.3 billion Indian citizens against COVID-19 by the end of the year, a mammoth task, while an estimated 90% of the adult population received one dose [2, 3]. As the number of daily doses administered has tapered off, officials underscore that decreased public demand and *not* supply issues are driving the slowdown [3]. The Indian Government approved AstraZeneca’s “Covishield” vaccine and the locally produced “Covaxin” vaccine, which required two doses to be administered 12–16 weeks (Covishield) and 4 weeks (Covaxin) apart [4]. Among the vaccinated, roughly 1 in 3 have not received a second jab and are not fully protected [5], compromising the success of the campaign. The disparity between the 1st and 2nd doses is the largest in the world, exacerbated as the demand for vaccines further declines.

### Vaccination in Odisha

Vaccine distribution is largely decentralized and varies within the different states in India [6]. Approximately 45.5 million people reside in the state of Odisha where the disease burden has been comparatively low, with 1 million confirmed cases and 8,400 deaths reported since the start of the pandemic [7]. While COVID-19 vaccine confidence data in Odisha is limited, a recent study of fewer than 400 respondents did find support for mandatory vaccination, which countered the belief that natural immunity would be more effective than a vaccine [8]. Focusing on the early stages of the vaccine campaign rollout, vaccine hesitancy was lower in Odisha than in many other Indian states, with less than 25% of the respondents unwilling to get vaccinated against COVID-19 [2]. The Odisha health department reported that 95% of 18-year-olds and above have received their first vaccine dose, but of those who have received their first dose, 92% are due for a second dose. It is worth noting that in India there may be a 14-week spread, depending on the particular vaccine being administered, between the first and second dose that might account for the disparity between first and second dose uptake [8]. The combination of high COVID vaccine initiation, but low COVID vaccine completion – when coupled with the low burden of disease in the state – makes Odisha an ideal environment to explore possible vaccine complacency and hesitancy around second dose completion.

Panda, et al., who studied vaccine acceptance in Odisha, found that the majority of their sample believed the vaccine to be safe, but also found variations in attitudes regarding immunity that follows infection as opposed to vaccination. Additionally, the researchers found two major barriers to vaccination: safety and awareness [9]. Young adults, who may have a greater sense of invulnerability than their elders and a life view that is focused more on the here and now as well as an accompanying distrust of authorities, are especially difficult to reach through traditional communication channels, especially when the messenger is an authority figure [10]. A recent study demonstrated how intergenerational relationships were leveraged to influence older adults’ vaccine uptake by communicating with younger participants through usage of social media [11]. The study, directed at marginalized communities, was based on the premise that young people are heavier users of social media and therefore are more likely than older people to engage with vaccine-related content.

While initial vaccination rates are reasonably high in Odisha, there is the issue of moving residents of all ages through the vaccine funnel from one to two doses of the vaccine and as they become available, a booster jab. As there may be low-risk perception regarding COVID-19, along with other factors like misinformation, there may be a lack of motivation to become fully vaccinated and a corresponding lack of interest in motivating others—friends, co-workers, and family members—to get fully vaccinated [12]. In addition, as there may be distrust of authority figures among young adults, those who deliver the message are of paramount importance regarding the acceptance of messaging instilling confidence in COVID-19 vaccines [13]. Also of importance is the context of the message promoting vaccine acceptance; the typical government official directly addressing an audience simply does not work with this population.

### YouTube as public health delivery system

In the digital age, peer-to-peer communication through mega- or nano-influencers may be more effective in getting the message across to targeted audiences. Thus, social media messaging operates best on a lateral plane with opportunities to communicate and spread information (and misinformation) through weak ties, as the strength of weak ties theory suggests people may be more influenced by those outside of their immediate social network than those with whom they have close ties, like family members [14]. The open structures of social media allow for influence to flow across networks in a scale-free manner in which actants, identified as nodes in the social network, have the potential to influence attitudes and normative behavior. YouTube as a dynamic visual medium has unique qualities to create an environment of indirect learning when it comes to public health messaging. There is no shortage of public health or more specifically vaccine awareness information on various social media platforms, but research to date suggests that while changes in attitudes may be accomplished, there has been no evidence of vaccine uptake [15]. The present study represents an opportunity to evaluate YouTube vaccine videos based on their spreadability (virality) and audience engagement.

Young adults (defined as those between 18 and 35 years of age) in Odisha are generally tech savvy and use their mobile phones to access popular social media platforms like Facebook and YouTube and messaging platforms like WhatsApp. In a study of social media strategies regarding vaccine acceptance, Limaye, et al. found that two key values regarding decisions related to vaccine acceptance were framing of the message and who delivers the message [16]. Li, et al. maintain that YouTube has not been utilized as an effective tool in health crises like Zika, H1N1, or Ebola, but the platform has great potential depending on the reputations of the sources delivering the message as well as the ability to work against the virality of misinformation [17]. Social media, because of its widespread use in India, can play a vital role in enhancing vaccine confidence. Penetration of social media platforms like YouTube (89%) and Facebook (76%) is deep among the target population (18–35 years of age) and secondarily their family and peers [18].

### Message design

While direct appeals from authority figures may fall short, there is evidence that young adults do desire to protect the wellbeing of others, in particular older family members whom they see as particularly vulnerable to COVID-19. Therefore, while young adults may be less motivated to get fully vaccinated themselves, there are indications that they may be driven by their desire to protect and act on behalf of others around them to encourage vaccination. Moyer-Gusé et al. studied the use of humorous versus non-humorous message design regarding Measles, Mumps and Rubella (MMR) vaccines [19]. While their messaging was directed toward parents, these researchers found that a satirical message reduced vaccine resistance and hesitancy. Their research concluded that a two-step flow approach allowed their messaging to effectively reach younger people through social media, who in turn were able to influence their elders, and was more impactful than trying to reach older people directly.

With the production of entertaining YouTube videos, we seek to enhance COVID-19 vaccine confidence among young adults. The approach to messaging is indirect, by encouraging young adults to serve as vaccine ambassadors, and encourage them to promote vaccination among friends and family, among others. As a result, we anticipate their own confidence towards becoming fully vaccinated will be enhanced. Beyond message design, YouTube’s algorithmic system plays a significant role regarding which videos are recommended to viewers, as there are YouTube recommendations, notifications, channel pages, and subscriptions that expose users to particular videos that also might show up on their “home” screen. As YouTube plays an increasingly significant role in disseminating health information [20] including information related to COVID-19 vaccination, based on our understanding of the nature of vaccine acceptance in Odisha, India, this research seeks to answer the following questions:

- RQ1: What role does YouTube’s algorithmic system play in the developing network that surrounds vaccine acceptance videos?
- RQ2: What are YouTube video viewers thinking and expressing based on their comments to the videos?
- RQ3: How might comments on the posted videos be categorized in terms of emergent themes and information?

## Materials and methods

This research evaluated two COVID-19 vaccination acceptance videos, viewer comments, various metrics, network detection, and comment sentiments. Extensive pre-planning took place prior to launching the two YouTube videos that included the production and testing of eight videos based on a 2 (appeal: individualistic or collectivistic) × 2 (tone: humor or non-humor) × 2 (protagonist gender: male or female) between-subjects design. For each video, 1–2 min in length, a male or female protagonist delivered the key messaging. Feedback on the scripts from a community advisory board of 11 individuals was incorporated, and the videos were produced in the Odia language. Further details about the study protocol are described in detail elsewhere [21]. As part of the formative research, that informed the creation of the videos, individuals were recruited to participate in an online survey questionnaire that addressed attitudes toward vaccination, vaccine behaviors, and vaccine intentions. 2,349 respondents completed the questionnaire that had demographic information, and beliefs about vaccination and is mentioned in detail elsewhere [22].

Two videos that represent opposing approaches based on the following criteria: male/individualistic/humorous (video#1); and, female/collectivistic/non-humorous (video#2) were selected and uploaded for dissemination to the Swasthya Plus Network on YouTube (https://www.youtube.com/c/SwasthyaPlus) [23, 24]. A social media campaign to promote the videos was based on organic search engine optimization (SEO) and pushed through WhatsApp messaging, important for enhancing discovery and engagement with the videos. Social media analytics software analyzing opinions expressed in public is becoming more widely used in the social sciences and was used in this project, to analyze COVID-19 vaccine acceptance through YouTube data. In addition to sentiment polarities – negative, neutral, or positive – algorithms are utilized to generate insights through community detection between users. Our research is based on both the analysis of YouTube comments and the networks that form around communities of users and is an approach that is meant to complement standard social science approaches.

In addition to standard metrics, we were interested in learning how viewers use these videos for sense-making purposes considering how videos traverse channels to reach viewers and the dramatic qualities that generate engagement. In order to understand this practice, we used YouTube Data Tools Channel Network Module (https://tools.digitalmethods.net/netvizz/youtube/mod_channels_net.php) to investigate the relationship between channels and the two video IDs. The module generates a network of channels in which a video from one channel points to the video of another channel, in which case an edge is created; the more times that appears, the more weight the connection gets. We analyzed the videos based on the following metrics: how long each video was viewed or watch time, average percentage viewed, average view duration, engagement, unique viewers, and viewer demographics. To analyze the channel and comment networks, we utilized YouTube Data Tools to extract the data from the two videos [25]. The tools provide a CSV file of the comments and a second graph file (GDF) for visualizing the network. The CSV file, including the comments, was uploaded to Communalytic, software that analyzes those comments for sentiment [26]. As necessary, the team in India translated the comments from Odia to English. Their translation considered culturally unique language. After the comment network was translated, a sentiment analysis was conducted using machine learning (ML) that identifies the polarity of comments as either negative, neutral, or positive. The graph file was uploaded to Gephi [27] open-source data visualization software that creates a map of the comment network allowing the researchers to see how the messaging in the videos is directed within the network of comments and how clusters are organized within the viewer community (with particular interest in who is influencing the network), which in network analysis are referred to as “hubs” and “bridges” that serve as community connections. Data based on the network of channels associated with our two seed videos provided additional visualizations.

## Results

This section describes the result of applying our methods to the two COVID-19 vaccination acceptance videos, as described above. We begin the analysis by comparing the metrics for the two videos (see Table 1).

**Table 1:** Metrics of 2 YouTube videos (February 2 – March 4, 2022)

YouTube Metrics	Video #1 – Feb. 4 Male/Ind./Comic (Protagonist Somanath)	Video #1 –March 4	Video #2 – Feb 6 Female/Coll./Serious—Mini	Video #2 –March 4
# of Views	1,510	1,628	5,700	7,500
# of Likes	93	84	81	217
Watch time (seconds)	20.3	21.5	68.4	101.2
Average Percentage Viewed	42.2	41.8	37.9	45.5
Average View Duration	0:48	0:47	0:43	0:48
Engagement	27,452		27,452	
Impression	27.500	34,000	78,600	
Who is watching (demos)	31.3 Fem. /	31.3 Fem./	40.3 Fem. /	31.Fem./
	68.8 Male	68.7 Male	59.7 Male	68.7 Male
Comments	25	31	11	149

These standard YouTube metrics are based on a 30-day time frame (February 4- March 4, 2022) in which SEO practices were used to organically boost the views, which was successful in “seeding” the videos in the channel network. As the two videos were placed on the Swasthya Plus health channel with over 450 thousand subscribers, we expected viewer reach would be significant. While 13,200 respondents viewed Video#2 for an average 84.8 seconds, 3,138 viewed Video#1 for an average 20.9 seconds.

### The Channel Network

To answer RQ1, regarding the role that YouTube’s algorithmic systems play in the developing channel network that includes or excludes vaccine acceptance videos, we investigated the channel network into which our two vaccine advocacy videos are connected. Algorithms play a role in serving media content to users; as such we seek to open the “black box” to understand how these algorithms moderate interaction. Through digital tracing, we can map the patterns of the ways by which people link to the videos and one another through community detection. The largest digital media platforms, for example, have recommender systems. These “delivery” systems determine what is likely to be seen as well as what is likely to be excluded. Sometimes what is seen is at the discretion of the user when they become a subscriber to a channel. At other times, the platform utilizes the user’s history to serve them content. The network that is formed is a product of the YouTube recommender system, which is a common form of machine learning that operates behind the scenes of Facebook, Twitter, and YouTube’s “suggested videos.” The traffic sources are reflective of user subscriptions, among other potential sources (see Table 2).

**Table 2:** Description of how viewers found the videos

Traffic Source	% Of Views Video #1	Views Video #1	% Of Views Video #2	Views Video #2
Recommendations	37.7	570	58.3	3,300
Home	30.4	459	48.9	2,800
Up Next	7.4	111	9.4	534
Notifications	29.6	447	18.3	1,000
External	14.3	216	9.5	537
Subscriptions feed	7.7	116	7.2	410
Other	10.5	159	6.6	373

Understanding how the two videos operate within that larger YouTube system and how they are linked to other video channels based on the YouTube algorithm forming a channel network is important for health communicators utilizing social media to communicate about vaccines as well as other health-related issues. Regarding message design, we were aware from previous experience to craft headlines and descriptions to avoid getting caught up in the web of exclusions by the recommender system.

**Fig. 1:**
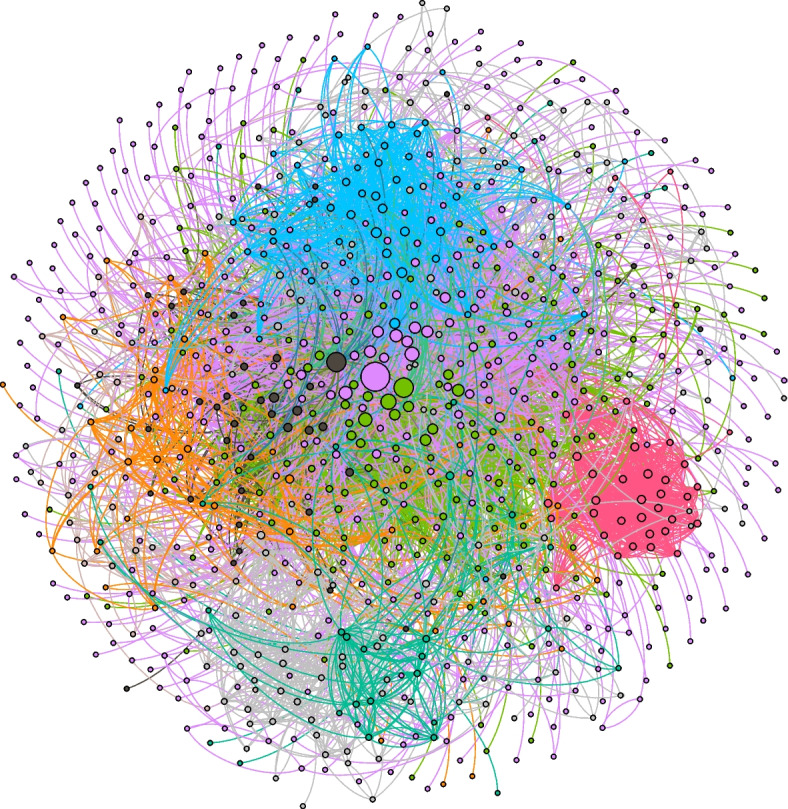
Shows the channel connections based on two video seeds

Figure 1 represents a visualization of related videos based on a measure of modularity, which represents the network fragmentation as it develops into distinct communities or clusters. Crawl depth is used in the search to see how far the two videos, referred to as “seeds” extend. For this analysis, we selected a crawl depth of 2, which yielded 2,517 video connections. The GDF file was uploaded to Gephi open-source data visualization software for analysis. There were 878 nodes representing the seed channels and 6,816 edges, which represent the connections between the nodes. The measure of modularity in this case is 0.638 and communities or clusters are represented by the different colors in the visualization. Clusters in this case represent groups of nodes that share a commonality, connection, or tie with the two seeded videos. Modularity measures vary between 0 (lowest) to 1 (highest). For example, a modularity measure closer to 1 indicates clear divisions between communities, whereas values less than 0.5 suggest that the communities overlap more; the network consisting of a core group of nodes. The modularity measure for this network is in the middle range indicating a well-connected network. Additionally, the visualization demonstrates what may be called a homophily effect in which like-minded individuals (or in this case channels) that share beliefs and perhaps behaviors come together as the different colors represent those interests. While we cannot determine exactly what brings these nodes together, channel subscriptions, and in that, an interest in health information, may partially explain the connections, along with YouTube’s recommender algorithm based on what videos a user has watched previously.

In addition, Fig. 1 shows the channel connections based on two video seeds resulting in a network of 878 videos, where each node represents a single channel with connections to our two seed videos. Following the principle of homophily, we expect similar content would cluster around interest in vaccine acceptance. The larger the cluster, the greater the strength of the node, meaning that the node is a central figure in the network and based on power law, is likely to be a driver of communication, calculated as a high degree, within the network. As indicated in the color legend below, approximately 42% of the nodes represented by the fuchsia color are connected to Swasthya Plus Odia, the home YouTube channel where the two videos are located. The central node in the green cluster is the Sidharth TV network, a general entertainment TV channel in Odisha. In the blue cluster Odisha Digital, a youth-oriented Odia channel is central and serves as a hub, and the central node in the black network is Nandighosha TV, a multi-platform channel on television, the internet, and mobiles.

**Fig. 2:**
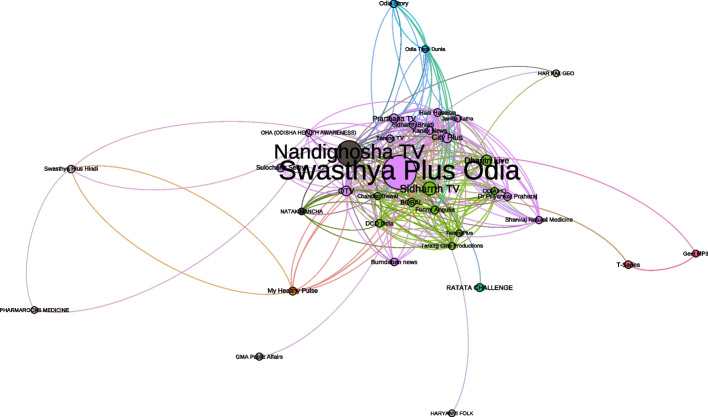
Depicts the network based on betweenness centrality

Next, we wanted to visualize betweenness centrality in this network. Simply put, a node with high betweenness centrality has a large influence, based on the transfer of information through the shortest path, to other nodes. Figure 2 depicts the network filtered to identify those nodes with high betweenness centrality. As noted above, the Swasthya Plus Odia network, the Nandighosha TV network, and the Sidharth TV network provide the greatest connections across the shortest paths in the network map. The value of this approach is so that we can visualize the path from a channel to the video that viewers are recommended based on YouTube recommendations and subscriptions.

These visualizations hold implications for this social network made up of connections to our two seeded videos. The higher betweenness centrality depicted in the larger colored circles in the visualization reflects power because those nodes exercise control over the dissemination of these videos within the YouTube system. Nodes with higher centrality also have stronger ties within the network and may serve as bridges to other clusters within the network, recalling that the network is made up of different clusters representing different interests or routes to the videos. It is important to point out that the titles of the seed videos did not contain references to COVID-19 or vaccines to avoid the potential for YouTube to block the videos as the platform has been removing anti-vaccine videos and it is possible that pro-vaccine informative videos might be caught in that process by the platform.

### The comment network

**Fig. 3:**
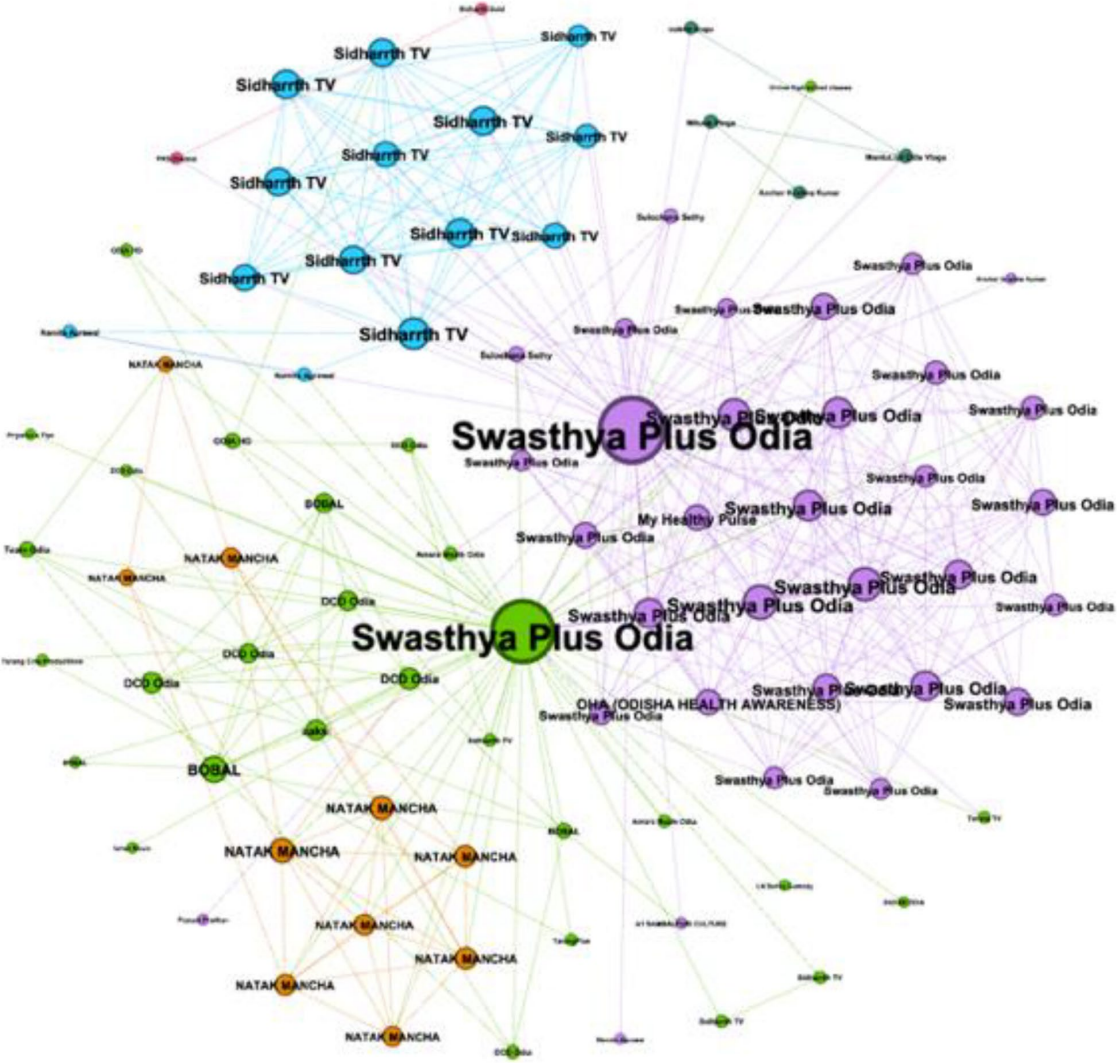
Visualization of the related YouTube comment network

To investigate RQ2 regarding the comments on the YouTube videos, we first looked at the comment network, similar to the approach taken in the presentation of data regarding the channel network above. We utilized YouTube Data Tools Video Network Module to retrieve information regarding the relationship between the two videos and the connected network of channels (https://tools.digitalmethods.net/netvizz/youtube/mod_videos_net.php). Important to this analysis, we were able to identify the key genres, like news channels or comedy channels operating in the comment network. Figure 3 is a visualization of the related YouTube comment network based on the two seeded videos. The two dominant circles in fuchsia and green represent the Swasthya Plus Odia network where our two videos are located.

This is a network with 88 nodes and 521 edges. The network diameter is 5 nodes, which refers to the length of the computed paths between all pairs of nodes in the network. The connection that could potentially exist between two nodes (regardless of whether it does) is reflected by the density measure of 0.68 and the average degree (5.92). The modularity measure is low at 0.491, which suggests this is a highly connected network.

**Fig. 4:**
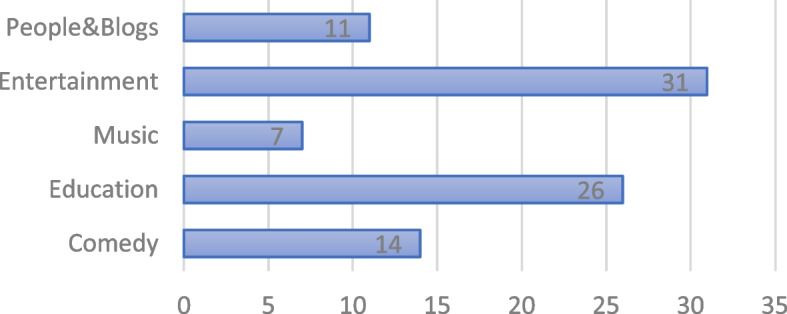
Video channel genres

Figure 4 is a list of channel genres to which the two video seeds are connected. The channel genres fall into five categories with one-third of the nodes being entertainment channels, followed by education channels at 26%. Fourteen percent of the nodes are comedy channels, people, and blog channels at 11% and music channel nodes at 7%.

Of interest is that of the connected channel genres, there are no health channels other than the Swasthya Plus Odia, which is itself a health information channel. The connections between the two videos and these other channels have implications for the total number of views and level of engagement with the two videos. In other words, a health-oriented video that is seeded into these other genres is important to health communication programs that seek to understand how network connections can facilitate communication of health-oriented information beyond the immediate channel in which the video is embedded.

### Comment network of user interaction

To further investigate RQ2, we looked at discourse and user interaction as depicted through visualizations of the relationships that form within and around our data set. As we have used this network analysis to look at the recommender system above, we turn to a closer look at the comment network of our two COVID-19 vaccine acceptance videos. The comment threads allow the researcher to look at influence within the conversations based on graph diameter, node degree, and graph density. These statistics indicate the shortest paths between two nodes, how close those nodes are to one another, and who the influencers are that may be serving as “hubs” in the network. Hubs in this sense, foster communication, and it is possible to identify those commenters who are serving as hubs to better understand who they are and the role they are playing within the network. The videos initially elicited a small number of comments upon launch (see Table 1), and on February 12, 2022, a contest was launched to boost the level of comments on the second video, “What is Mini’s Gift?”, which is what the following analysis is based upon (YouTube Video #2, 2022).

**Fig. 5:**
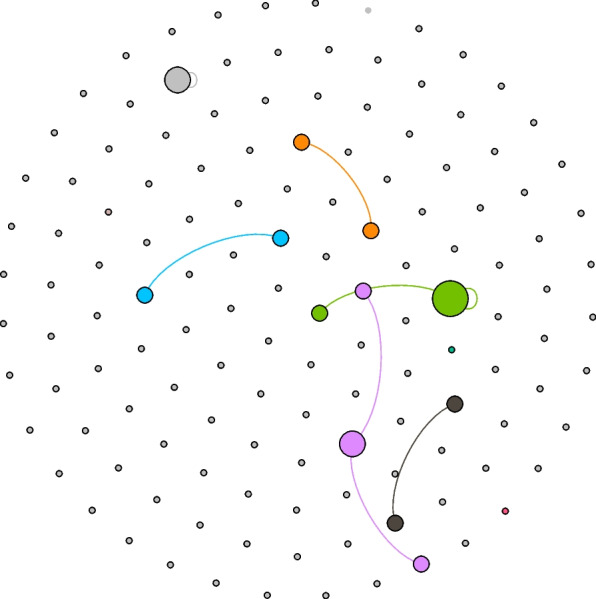
Visualization of the network of comments

The video titled “What is Mini’s Gift?” comment network is comprised of 134 nodes representing comments and 8 edges (Fig. 5). Modularity is 0.778, suggesting this is not a well-connected network. Visually, one can see there is little interaction between comments and replies, as indicated by the few edges in this network. It is common in the YouTube comment stream for there to be a lack of or limited number of replies to comments. Sometimes, interaction does not manifest as a reply to a comment. However, there are opportunities to “like” the video and to “share” the video, which are indicators of interaction.

### Sentiment analysis of youtube comments

To answer RQ3, regarding the sentiment of comments, we turned to sentiment analysis, also known as opinion mining, to classify comments into three types: strong positive, negative, or neutral. As with varying opinions regarding COVID-19 vaccine acceptance, we would anticipate that viewers of the videos would elicit strong responses. Generally, comments reflect people’s thoughts, feelings, and intentions to behave. YouTube, a widely used social platform, attracts views every day, which makes crawling users’ comments a potentially ongoing activity since the videos were posted on February 4 and monitored until March 4, 2022. In this study, those comments taken together represent the virtual collective consciousness, that is the direction of thought around COVID-19 vaccines in relation to the posted videos. This approach to social media research provides real-time monitoring of commenters’ attitudes and serves to supplement more traditional approaches to research. We used the Sentiment Analysis Module of Communalytic for the sentiment analysis of YouTube comments. This module detects the polarity of posts in the dataset of comments [26]. It categorizes comments as negative, neutral, or positive sentiments. To calculate the scores, the module uses two types of analysis VADER (Valence Aware Dictionary for Sentiment Reasoning) and TextBlob. The comments were translated from Odia to English for processing; comments in other languages were skipped. Table 3 is a sentiment analysis of 62 of the 104 posts. Based on the VADER results 80% of the comments were positive, whereas TextBlob determined that 62% were positive. Fewer than 2% of the comments were negative, and VADER determined that almost 18% of the comments were neutral, TextBlob categorized 37% as neutral.

**Table 3:** Sentiment analysis of 62 out of 104 comments

	# of Posts	Negative Sentiment	Neutral Sentiment	Positive Sentiment
VADER (English/EN)	62	1 (1.61%)	11 (17.74%)	50 (80.65%)
TextBlob (English/EN)	62	0 (0.00%)	23 (37%)	39 (62%)

**Fig. 6:**
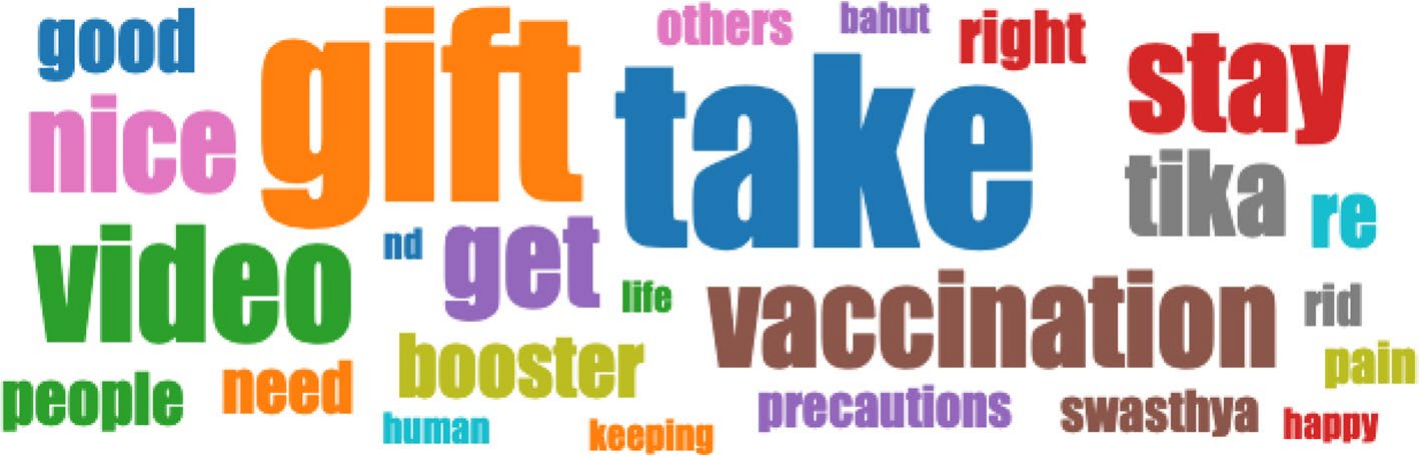
World cloud indicates how often a word is used in the comments

We raise the question regarding why some words, or for that matter images, show up rather than some other language or images. To that end, a word cloud represents a means of visualizing language utilized in the YouTube comments. Figure 6 represents a word cloud of the most often-used words in the comments with a larger depiction indicating that the word was used more often than others. When we see words like “gift” or “take” dominating the cloud, we can interpret the meaning of the messaging which is further amplified by the other positive words that make up the comment cloud, like “nice”, “vaccination”, “booster”, and “get”.

To further investigate RQ3, in addition to analyzing sentiment, a thematic analysis of the comments was conducted from which five broad categories emerged: sharing beliefs, sharing knowledge/information, safeguarding self and community, personal experience, and reactions to the video. Table 4 presents a sampling of YouTube comments adds qualitative evidence of the positive sentiment described above.

**Table 4:** Four Themes of YouTube comments

Theme	Comment Example
Sharing Beliefs	*Corona virus is not going to go away from this world. So we have to be prepared for the virus in such way, that the virus can't affect us. So right now the only cure for it we have is vaccine and masks* *We all should get completely vaccinated. Mini is conveyeing the right message* *Health is wealth* *The person who is healthy and safe both physically and mentally, only they can help create a healthy society. My best wishes to them*
Sharing Information	*Covid vaccine hesitancy is addressed in this video very deliberately. While developing the covid vaccine we take 100% precautions and safety checks before opening for human use. We all need to understand the seriousness of the situation and take 2 nd dose of vaccine. It is also advisable for the exposed groups to take the third dose which is booster dose after consulting with physician. Very dynamic initiative from Swasthya plus team. Kudos* *Sanitizer mask and hand* *"Our government is not our enemy. It has been raising awareness about the epidemic. For this reason, they have been spending billions of rupees from the exchequer to get back to normal." Follow the Covid guidelines (government) for your benefit and your community* *Everyone needs to have a sense of responsibility as citizens. We must have a strong faith and belief in the science of medicine. According to the rules of the government, vaccines need to be taken at the right time. As a result this whole society will stay safe*
Safeguarding self and community	*We all should get vaccinanted (sic) completely, we will stay safe and there will be less infection in our country. We have to make people understand* *Mini has given a lesson to the family, stay healthy and safe, keep others healthy and safe and it can protect the world from the covid pandemic. Because, every live and every human is valuable* *Take vaccine, stay safe and make other safe* *We all should get vaccinanted(sic) completely, we will stay safe and there will be less infection in our country. We have to make people understand* *The only way to get rid of corona is vaccine. It is really important to take both the doses of the vaccine. Because if we stay healthy and safe, we can keep our family healthy and safe and then our state, country and the then we can get rid of this virus from the whole world. Let's get vaccinated and get rid of corona and create a society as it was earlier. We need to spread awareness about corona. I am saying it again get vaccinated and remove corona*
Personal Experience	*Vaccination is the biggest gift. I have got vaccinated, you guys should get vaccinated too*
Reactions to Video	*This is incredible!* *Thank u so much for share this video* *Nice video*

This qualitative approach provides insights into how viewers are thinking in response to or reacting to the video. The following comment stood out as it exemplified the impact that the messaging in the video was designed to elicit among viewers.

*“Who knew Covid19 would spread all over the world in days and take on such a terrible form. It took lives one after another for no reason at all, the pain of losing a loved one could only be explained by the person who went through this. This pandemic became so terrifying that the government had to take the decision of stopping the trade, commerce, transportation, and educational institutions. Which affected the common society a lot and nothing like what it used to be. The knowledge of Indian and the scientists from overseas helped create the vaccine for the prevention of the spread of the COVID infection. It was made available to the civilians for free and an order was issued by the World Health Organisation that instructed the general public to abide by the enacted law which was effective in each state accordingly. Those were wearing the mask while stepping out, maintain social distancing, go out only if needed and keep washing hands regularly. The public also followed it and though the COVID cases were starting to reduce everyone started to go out withought (sic) any reason. As a result of which the world is now suffering. Taking both the doses of the vaccine doesn't mean haat [sic] we are safe, the virus and the infection hasn't yet gone. The world needs proper awareness of how to prevent themselves from this pandemic. After taking the second dose one should get the booster dose as well. Follow the government's rules and help eliminate the epidemic from the world. As a medical student, my hope is that the epidemic will not last long. Only awareness can save our precious lives. Hopefully sensitive people will accept it”*

## Discussion

The World Health Organization (WHO) in response to the rise of anti-vaccine sentiment established a Strategic Advisory Group of Experts on Immunization (SAGE) to monitor vaccine confidence, vaccine complacency, and to consider the accessibility of vaccines on a worldwide basis [28]. Consistent with the goal of SAGE, our research goes beyond traditional approaches to deal with these vaccine acceptance issues to consider the time people spend on digital media, in particular social media, and the potential of platforms like YouTube to serve as a viable source to support COVID-19 vaccine acceptance.

COVID-19 vaccine acceptance continues to be a challenge for the world. This research project is particularly interested in vaccine acceptance in Odisha, India. The goal of this research project was to create communication based on appeals that are relatable to young adults and that inform and encourage them to serve as ambassadors to their friends, and family. Message design was of paramount interest, as research suggested that top-down messaging that might be heavy-handed in its approach may not work with this target population. Rather, we developed a more indirect approach, one based on ordinary people in relatable situations to deliver the key message in under 2-min. We found that the serious video with a collectivistic appeal performed better than the comedic video. This finding stands in contrast to other research that suggested that a more humorous/sarcastic tone would be more appealing to young adults. This finding emphasizes the need among health communicators to understand the local culture and setting to identify appeals that are likely to be most effective in getting messages regarding vaccine acceptance across to target audiences.

The study also provided evidence of the importance of understanding effective communication goes beyond the content of messaging to consider the ways in which social media platforms operate. To that end, the visualizations of the channel network in which our videos reside indicate that YouTube videos do not exist in a vacuum. Rather, they are part of a subscriber and recommender network, which is important to disseminating the messages contained in the videos. While the two seed videos created connections with more than two thousand other videos, it was valuable to learn that these videos were not just located on health-oriented channels but were on news and entertainment channels that were not specific to health communication. Additionally, we were able to extract comments from one of the videos for sentiment analysis which found those comments to be mostly positive. While there were some neutral comments, the research found no negative comments. Finally, we did a content analysis of those comments in which five categories emerged. Examples of those comments provide additional insight into the reception of the messaging. While YouTube and other social media platforms are attempting to weed out COVID-19 misinformation, there remains a need to promote vaccine acceptance to those who are in most need of that information in ways that are most palatable, and through channels that get the messaging across to the right audience.

In this applied research project, we were interested in message design regarding COVID-19 vaccine acceptance and the impact messaging would have on the target audience of young adults in Odisha, India. While the study makes no claims correlating message design and vaccine uptake, the findings are indicative of the spread or virality of the two videos and their ability to garner and sustain attention. We selected two videos to upload to the YouTube platform, each representing a contrasting approach, but both offering pro-vaccine messaging. The videos varied in appeal, tone, and protagonist. This is an organic approach with no paid advertising to influence viewership, thus the measures of views, likes, and comments are reflective of the popularity of each video. The video with the collective appeal, and serious tone, with a female protagonist garnered more attention, that is views, than the alternative video that was based on a comedic tone, individualistic appeal, and male protagonist. In the interest of boosting comments, we used WhatsApp to encourage additional viewership, resulting in added comments upon which our analysis is based.

While there is no shortage of information from various sources that the vaccines are effective at preventing severe illness, research suggests people are not getting the second dose as it becomes available, making the virus difficult to contain. That being the case, the benefit of vaccination is to a great extent an individual choice rather than a collective benefit, which runs contrary to the popularity of the message in the serious collectivistic approach that initially garnered twice as many views as the comedic individualistic approach. As the gender of viewers was relatively even, it is not likely that the gender of the protagonist delivering the key message confounded this finding, however additional research would have to confirm this outcome. The popularity of the video with a serious tone and collectivistic appeal suggests to us that this approach should be advanced in future vaccine acceptance campaigns in Odisha, India. However, we cannot make a general claim about the effectiveness of the messaging in other geographic locations. Rather, our project emphasizes the need to go beyond standard metrics to understand not only the local culture when designing and implementing health communication campaigns but the platforms on which messaging is conveyed.

Though our research produced a total of 8 videos of varying combinations of appeals, tone, and protagonist gender, here we investigate message design in two of these videos. Future research should investigate the impact of the messaging design of other combinations of these videos. When considering creative approaches to vaccine acceptance message design, it is important to consider not only the creative content of the messaging but the system in which the messaging operates. Effective SEO along with the creative effort can mitigate the limitations of recommender systems, along the way ensuring that content does not inadvertently get caught up in exclusionary algorithms.

In this research, we have developed an approach that makes algorithmic outputs visible and offers a means to describe them through visual analysis and thematic analysis of the comments posted by viewers of the videos. Effective message design can facilitate communication of health-oriented information beyond the “home” channel in which a video is embedded. This is an important takeaway for health communicators designing vaccine acceptance social media campaigns. The positive sentiment expressed by those viewers in this study points to the countervailing force that the message design utilized in this applied research is an effective tool with the potential to positively impact attitudes, as our online lives become ever more intertwined with our offline decision-making when it comes to vaccines.

This study has several limitations. The results of this study are specifically reflective of the conditions in India in February/March 2022, approximately a year after the availability of the COVID-19 vaccines and not during the entire course of the vaccination campaign. The nature of YouTube varies greatly regarding how videos gain acceptance among larger groups of viewers, in other words, raising questions regarding how an audience develops over time. Some video viewership spikes quickly, while others remain at a low level of viewership and may spike at a later date. In other words, this research operated in the uncontrolled real-time world of YouTube. While the research was able to answer the question regarding the sentiment of comments, the number of comments was relatively small. Similar to video views, comments may over time increase, suggesting the need for ongoing monitoring of the video network. A limitation of network analysis is the inability to understand the strength of connections in the network. The size of nodes in the comment network is based on a measure of degree, which refers to viewers who are commenting (out-degree) or being replied to (in-degree). The analysis of the comments is based only on the views expressed by those who watched the videos or watched at least part of the videos and offered their commentary. The study is reflective of the comments of those who engaged with the videos, motivated by the messaging to offer their thoughts. However, everyone who watched the videos did not offer comments, so the analysis is reflective of a subset of views of the videos.

## Conclusion

Our study is based on the idea that vaccine acceptance depends on whether people feel that vaccines are effective, trust in government and other authoritative sources, and have access to vaccines. Furthermore, vaccine acceptance is not only an individual decision but one that may be collectivistic, that is a decision based on looking out for one’s family, co-workers, and community. Unfortunately, there has been a significant amount of anti-vaccine misinformation in circulation that confuses the issue. Our study shows that a video is an effective tool for spreading messaging in a visually engaging manner, using high production values and relatable characters to engage audiences. In this way, we hope to counteract negative thoughts about COVID-19 vaccination and enhance vaccine confidence in the process.

For public health communicators and other stakeholders with an interest in vaccine advocacy, this study suggests it is important to go beyond so-called vanity metrics, including shares, likes, etc. when measuring campaign outcomes. Rather, the social network analysis presented in this study allows researchers to dive deeper into, for example, the polarity of sentiment regarding, in the case of the present research, YouTube comments. Beyond comment analysis, the social network analysis approach creates opportunities to visualize the network of video channels with which the content is connected. As health communicators adopt social listening tactics, it is important, based on the findings of the present study, to understand that social media content operates within a larger techno-ecosystem. As such, videos placed on YouTube do not exist in a vacuum. Rather, they operate within the platform’s recommender and subscription systems and among other sources of traffic. In conclusion, these findings have implications for message design and dissemination regarding optimizing social media content to enhance vaccine acceptance.

## Data Availability

Data utilized in this study may be provided upon request from the corresponding author.
