# Correction: Social network analysis of COVID-19 vaccine YouTube videos in Odisha, India: mapping the channel network and analyzing comment sentiment

**DOI:** 10.1186/s12919-023-00267-w

**Published:** 2023-07-13

**Authors:** Neil Alperstein, Paola Pascual-Ferrá, Rohini Ganjoo, Ananya Bhaktaram, Julia Burleson, Daniel J. Barnett, Amelia M. Jamison, Eleanor Kluegel, Satyanarayan Mohanty, Peter Z. Orton, Manoj Parida, Sidharth Rath, Rajiv Rimal

**Affiliations:** 1 Department of Communication, Loyola University, Maryland Baltimore, USA; 2 School of Medical and Health Sciences, George Washington University, Washington, D.C. USA; 3 Bloomberg School of Public Health, Johns Hopkins University, Baltimore, Maryland USA; 4 Development Corner (D-COR), Bhubaneswar, Odisha India; 5 Wellflix Inc., Hillsborough, USA; 6 Swasthya Plus Network, Bhubaneswar, Odisha India

**Correction: BMC Proc 17, 9 (2023)**

**https://doi.org/10.1186/s12919-023-00260-3**

Following publication of the original article [1], the authors identified an error in the author names of Ananya Bhaktaram and Satyanarayan Mohanty.

The incorrect author name is: Ananya Bhakktaram

The correct author name is: Ananya Bhaktaram

The incorrect author name is: Satyanarayana Mohanty

The correct author name is: Satyanarayan Mohanty

The author group has been updated above and the original article [1] has been corrected.
